# The impact of climate change in wheat and barley yields in the Iberian Peninsula

**DOI:** 10.1038/s41598-021-95014-6

**Published:** 2021-07-29

**Authors:** Virgílio A. Bento, Andreia F. S. Ribeiro, Ana Russo, Célia M. Gouveia, Rita M. Cardoso, Pedro M. M. Soares

**Affiliations:** 1 Instituto Dom Luiz, Faculdade de Ciências da Universidade de Lisboa, 1749-016 Lisbon, Portugal; 2 Institute for Atmospheric and Climate Science, ETH Zurich, Universitätstrasse 16, 8092 Zurich, Switzerland; 3 Instituto Português do Mar e da Atmosfera, I.P., 1749-077 Rua C do AeroportoLisbon, Portugal

**Keywords:** Climate change, Climate-change impacts, Climate-change mitigation, Climate sciences, Environmental sciences

## Abstract

The impact of climate change on wheat and barley yields in two regions of the Iberian Peninsula is here examined. Regression models are developed by using EURO-CORDEX regional climate model (RCM) simulations, forced by ERA-Interim, with monthly maximum and minimum air temperatures and monthly accumulated precipitation as predictors. Additionally, RCM simulations forced by different global climate models for the historical period (1972–2000) and mid-of-century (2042–2070; under the two emission scenarios RCP4.5 and RCP8.5) are analysed. Results point to different regional responses of wheat and barley. In the southernmost regions, results indicate that the main yield driver is spring maximum temperature, while further north a larger dependence on spring precipitation and early winter maximum temperature is observed. Climate change seems to induce severe yield losses in the southern region, mainly due to an increase in spring maximum temperature. On the contrary, a yield increase is projected in the northern regions, with the main driver being early winter warming that stimulates earlier growth. These results warn on the need to implement sustainable agriculture policies, and on the necessity of regional adaptation strategies.

## Introduction

Climate change and agriculture are fundamentally interconnected worldwide^1–4^. Indeed, the former has laid significant challenges to the agricultural sector in the last decades and is expected to further amplify these challenges in the near future^5–7^. On the other hand, agriculture is a major producer of greenhouse gas emissions to the atmosphere and thus partially responsible for causing climate change^8,9^. To overcome this cause/effect problem, policy reforms must be applied at the sector, national and international levels^10^. Examples are the implementation and correct execution of the Paris Agreement (COP21 Paris) by the countries’ governments and the promotion of sustainable agriculture by reforming policies that stimulate unsustainable intensification and overuse of natural resources^11–13^. An additional burden related with intensive food production is that the world population is expected to significantly grow until the end of the century^14^, which creates adaptation problems concerning freshwater usage and food security^15–17^.

Wheat and barley are two of the major crops in the world used for food and drink production, being the nutritional basis for both humans and animals alike^18^. Hence, these crops are fundamental for global food security. Rainfed wheat and barley are key crops in the Iberian Peninsula, particularly in Spain (EUROSTAT, 2019), where their cultivation areas represent about 7% and 22%, respectively, of the total EU-28 areas dedicated to these crops. Indeed, according to the EUROSTAT report for 2019, Spain is the country with the largest barley cultivation area in the EU-28; and is the 5th largest wheat cultivation area (following France, Germany, Poland, and Romania) in the EU-28.

The Mediterranean region, and in particular the Iberian Peninsula, has been identified as prominent climate change hot spot^19,20^. Nevertheless, climate change impacts and hence their consequences are not evenly distributed in space^21^, with their magnitude varying from region to region. With the aim of assessing this local to regional spatial variability, experiments to dynamically or statistically downscale Global Climate Models (GCMs) were designed within projects such as the Coordinated Regional Downscaling Experiment (CORDEX)^22,23^. As a result, a number of studies were developed resorting to these projects with the aim of analysing impacts of climate change on precipitation and temperature during the twenty-first century in regions encompassed within the Iberian Peninsula. These studies have consistently projected a decrease in the mean precipitation, and an increase in extreme precipitation events^24–27^. Furthermore, other studies ascertain a significant increase of both maximum and minimum temperatures in all seasons and climate change scenarios^28,29^.

Multiple linear regression models are a relevant statistical tool used to model a given predictand by taking into account a set of key predictors^30–32^. These predictors may be selected by applying screening regression techniques, such as forward or backward stepwise regression analysis^33^. Such techniques have previously been employed to model past and future wine production from the Douro Valley in Portugal^34^, or to model wheat and barley yields in the Iberian Peninsula using drought indicators, such has the Standardized Precipitation-Evapotranspiration Index, and the remote sensing indices Vegetation Condition Index and Thermal Condition Index^35^. Additionally, several recent studies tackled the issue of wheat and barley yield fluctuations due to climate change, over different regions of the world^36–42^. Others, focused on the Iberian Peninsula, have studied the impacts of climate change on crop yields by using mechanistic crop growth models^43–45^, which require a high volume of input data, rigorous calibration, and often several in-situ observations^46^. Moreover, previous works for this region assessed the effects of projected maximum temperature extremes on cereal crops^47^, analysed the adaptation measures for managing wheat in a changing climate^48^, and studied how the diurnal temperature range and drought may affect wheat yields in the future^49^.

This work aims at expanding previous ones by assessing how climate change may affect both wheat and barley yields in the Iberian Peninsula. This is achieved by using a wide range of EURO-CORDEX RCMs simulated meteorological variables (such as maximum and minimum air temperatures and precipitation) representative of present and future climates, according to the Representative Concentration Pathways RCP4.5 and RCP8.5 greenhouse gas emission scenarios^50^. The relation between the meteorological constraining variables and wheat and barley yields is used to develop empirical models based on multiple linear regressions with the objective of projecting these yields in the future. Although the use of empirical relationships may not capture the biophysical aspects of crops, such models represent rather well the large-scale impacts of climate hazards and require lower computational costs than mechanistic modelling^51,52^. Hence, this work is divided in three main objectives:

1. To establish a relation between wheat and barley yields and climate variables such as maximum/minimum temperatures, and accumulated precipitation based on the CORDEX-Evaluation RCMs (forced with ERA-Interim).
2. To calibrate and validate regression models using predictors from the CORDEX-Evaluation RCMs, by comparing observed and modelled wheat and barley yields.
3. To apply these regression models to CORDEX-Historical and CORDEX-Future RCMs, according to RCP4.5 and RCP8.5 scenarios, with the aim of studying and understanding future yield changes.

This work aims at assessing how wheat and barley yields may evolve if everything except climate remains unchanged. Ultimately, this study may be used as a helpful tool for decision-makers in the agriculture sector in the Iberian Peninsula, and hopefully it may warn on the need of implementing revised policies to immediately adapt and mitigate.

## Results

### Wheat and barley yield relation with temperature and precipitation

**Figure 1:**
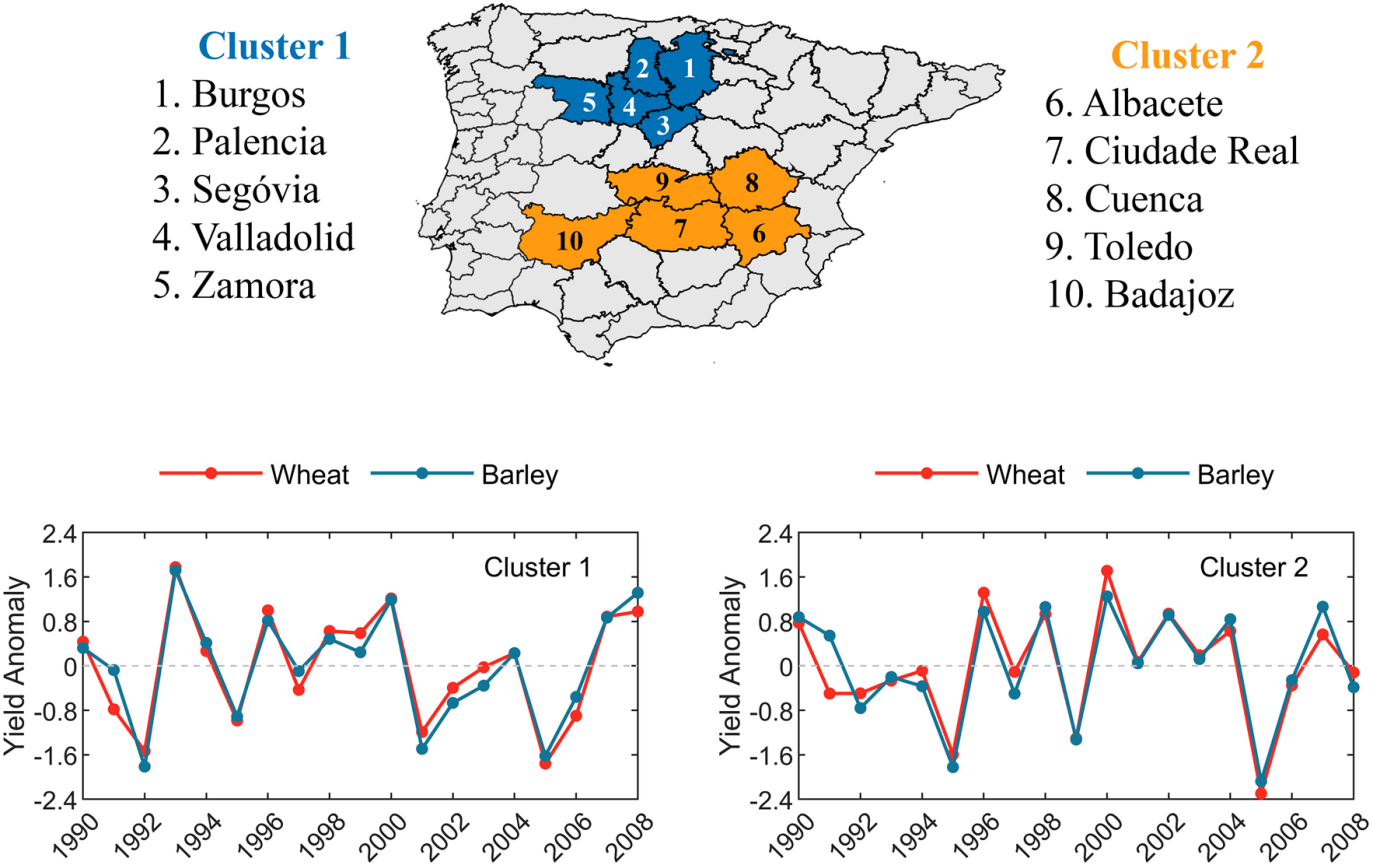
(top) Map with provinces that encompass cluster 1 (blue) and cluster 2 (orange); (bottom) Standardized detrended yield anomaly (μ = 0, σ = 1) of wheat (red) and barley (blue) for cluster 1 (left panel) and cluster 2 (right panel).

**Figure 2:**
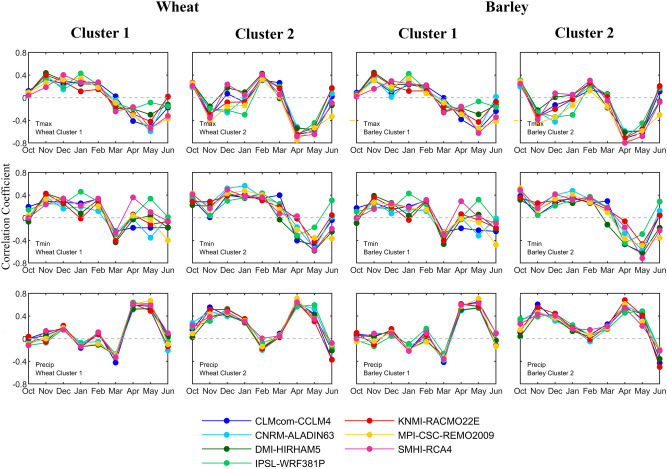
Correlation between standardized detrended yield anomaly of wheat (left panels) and barley (right panels) and CORDEX-Evaluation models standardized detrended precipitation (bottom), Tmax (top), and Tmin (middle) for cluster 1 and cluster 2.

Two clusters in the Iberian Peninsula mainly composed by rainfed wheat and barley are selected to perform this study (Fig. 1). Correlations between observed wheat and barley yields and maximum air temperature (TX), minimum air temperature (TN), and accumulated precipitation (PR) simulated by the seven CORDEX-Evaluation RCMs show an overall good agreement (Fig. 2).

The case of PR is the most striking, showing a large consistency among RCMs for wheat and barley over the two clusters. Moreover, the dependence of cereal yields on climatic variables reveals a contrasting behaviour between the two clusters. In cluster 1, positive anomalies of precipitation in April and May are the most important contributors to wheat and barley yield, while positive (negative) precipitation anomalies in March embody a negative (positive) effect in these yields. In cluster 2, positive anomalies of precipitation in April and May provide an important contribution to the productivity of the two cereals, albeit with a decrease in relevance of May precipitation when compared to cluster 1. Furthermore, winter precipitation proves to be an important factor to harvested yield, with November (wheat and barley) and December (wheat) showing large positive correlations.

On the other hand, TX and TN correlations with observed wheat and barley yields show larger variations dependent on the selected RCM. These are most prominent over cluster 1 (northern), where TX and TN show contrasting correlations in April and May, depending on the chosen RCM. The most notable differences are between IPSL-WRF381P TX and TN (correlations of about -0.1 and 0.4) and CNMR-ALADIN63 TX and TN (correlations of about -0.6 and -0.3) with both cereal yields. Due to inter-RCM differences, it is not as simple to define the most important yield-driving factors as with PR. In general, in cluster 1, warm (cold) winters and mild (warm) springs are the most favourable (unfavourable) to wheat and barley growth. While for the case of TN, the most consistently important month among RCMs is March, where mild (warm) minimum temperatures have a positive (negative) contribution to cereal yield. In cluster 2, TX in April and May, and TN in May show a large relation with both wheat and barley yields (with correlations up to -0.8). Thus, for each cluster and cereal a stepwise regression method is applied to select the most relevant predictors for the various CORDEX-Evaluation RCMs.

### Development of regression models

**Figure 3:**
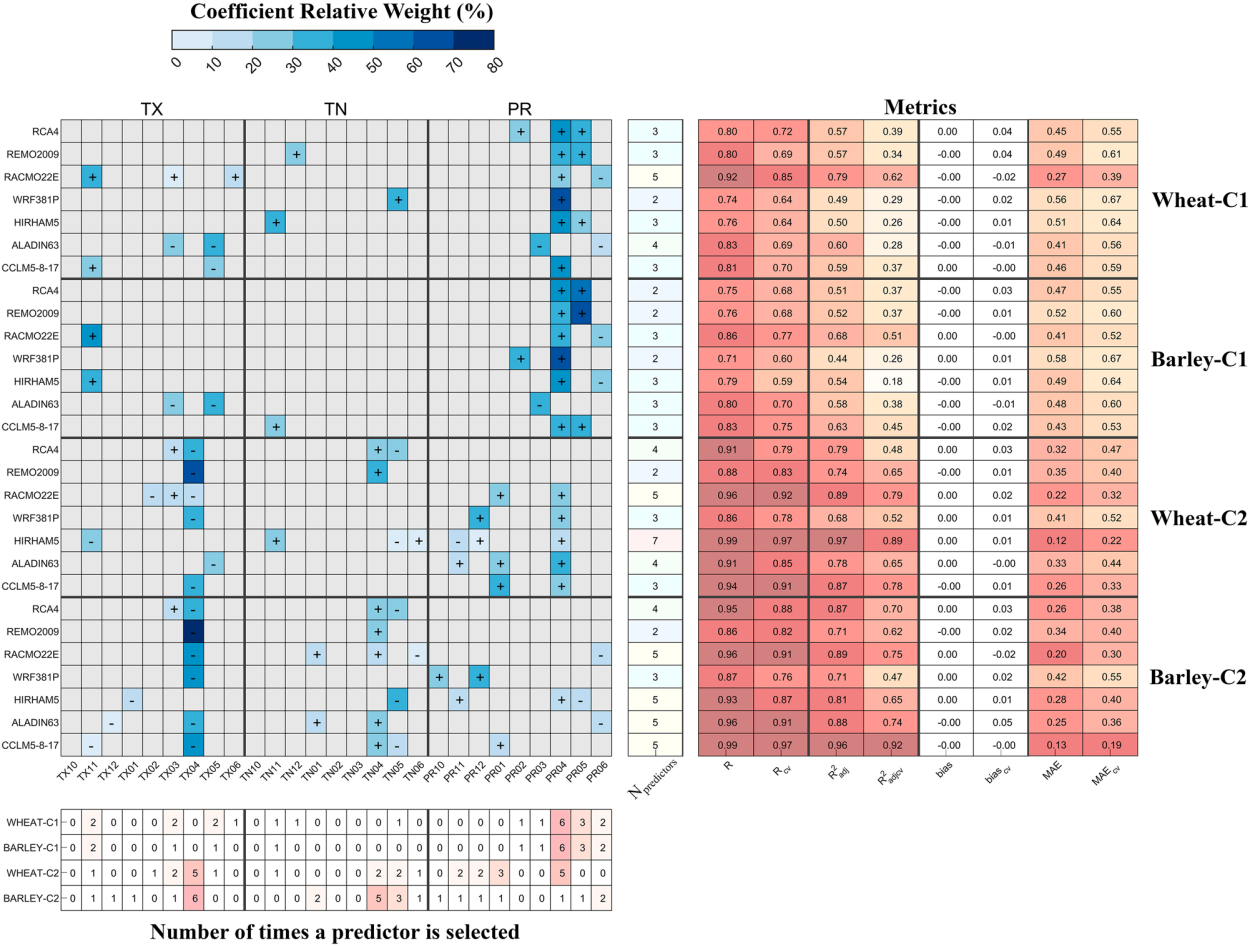
Representation of the selected predictors (displayed in tones of blue) for each CORDEX-Evaluation RCM. The matrix is divided into 9 blocks corresponding to TX, TN, and PR predictors from October to June (left to right), and the 7 RCMs for wheat and barley in cluster 1, and wheat and barley in cluster 2 (top to bottom). Tones of blue indicate the relative percentage of the coefficient associated to a given predictor and sum to 100% for each RCM. Symbols ‘+’ and ‘-’ specify if the coefficient is positive or negative, respectively. The bottom table shows the number of times a predictor is selected by cereal and cluster. The single column table at the right of the matrix indicate the number of predictors by RCM, cereal, and cluster. The table on the right displays the metrics associated to the comparison between cereal yields observed and predicted with regression models before and after cross-validation (cv): correlation R, ${R}_{ajd}^{2}$, bias, and MAE, in order from left to right.

Selected predictors—one set of predictors for each CORDEX-Evaluation RCM—to forecast wheat and barley yields over the two clusters are shown in Fig. 3. Different shades of blue indicate the linear regression coefficient relative weight, and the symbols ‘+’ and ‘-’ indicate a positive or negative coefficient, respectively. As an example, let us consider the predictors that are selected to forecast wheat yield over cluster 1 with CCLM5-8-17 model (evaluation): TX11, TX05, and PR04 (November and May TX, and April PR). The linear regression model may be written as ${Y}_{wheat}={a}_{1}TX11-{a}_{2}TX05+{a}_{3}PR04$, where ${a}_{1}, {a}_{2}, {a}_{3}$ are the regression coefficients, having relative weights of 27%, 30%, and 43%, respectively, for the exemplified case.

Although different sets of predictors are selected for each combination of RCM/cluster/cereal, some general patterns are found. For cluster 1, the most relevant predictors are PR04 (for 6 out of 7 RCMs, both cereals) and PR05 (3 out of 7 RCMs, both cereals), which is in agreement with Fig. 2.

In the case of cluster 2, wheat and barley predictors are not similar, with TX04 (5 out of 7 RCMs) and PR04 (5 out of 7 RCMs) selected to forecast wheat, whilst TX04 (6 out of 7 RCMs) and TN04 (5 out of 7 RCMs) are selected for barley. Furthermore, precipitation in winter seems to have some degree of importance in forecasting wheat (5 out of 7 RCMs selected: November (2), December (2), and January (3)), which is on par with results from Fig. 2.

In general, linear regressions are characterized by 2–5 predictors, depending on the RCM. The exception is DMI-HIRHAM5, which regression is composed of 7 predictors. However, tests show no conspicuous overfitting. Moreover, correlations after cross-validation range between 0.59 (explaining 18% of the variance) and 0.97 (explaining 92% of the variance). Notably, cluster 2 shows smaller differences (MAE ranging from 0.19 to 0.55) between observed and forecasted yields than cluster 1 (MAE ranging from 0.39 to 0.67).

**Figure 4:**
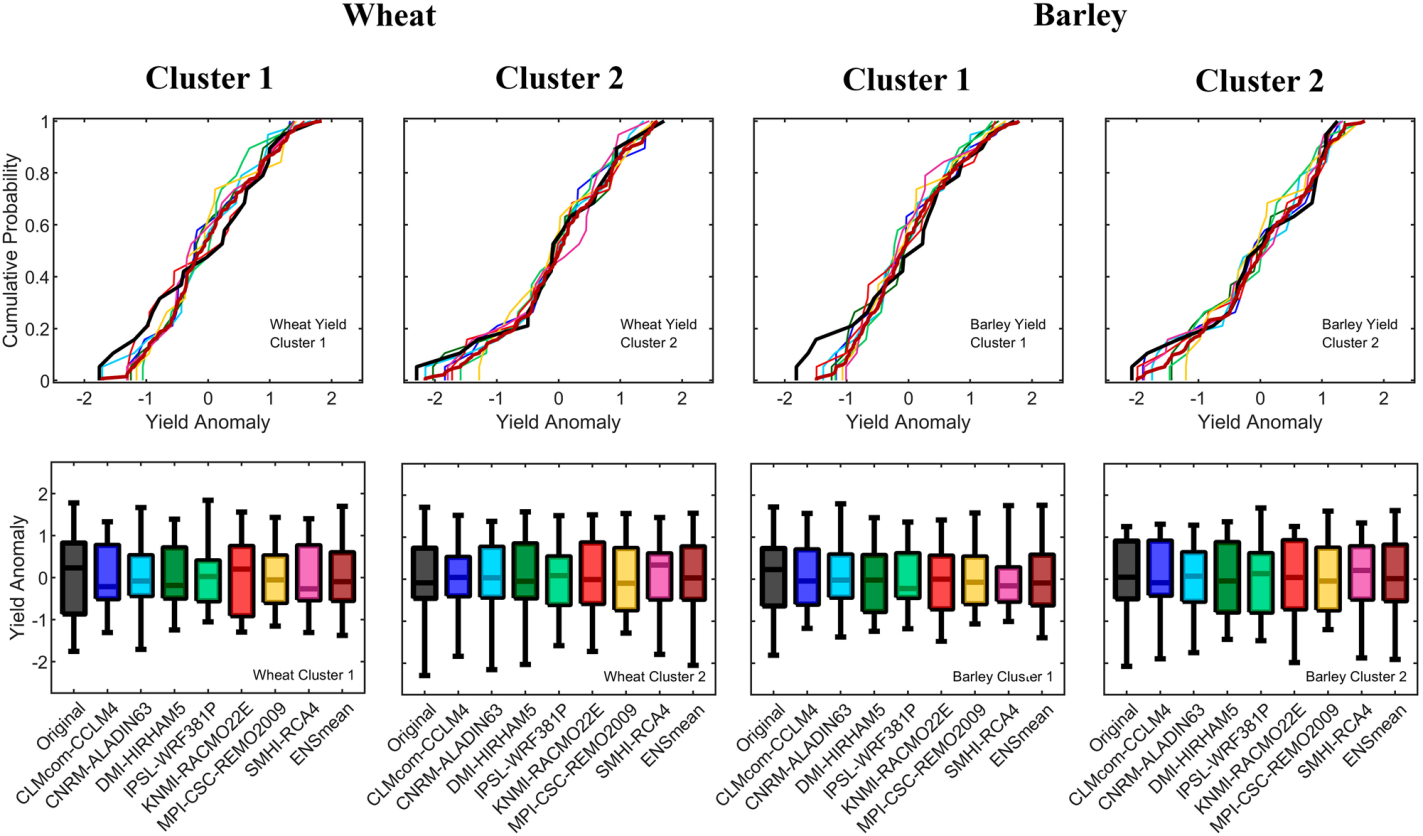
Comparison between distributions of yield anomaly in the form of ECDFs (top) and boxplots (bottom) for the 7 CORDEX-Evaluation models, the multi-model ensemble yield mean, and the observed values. Boxes range from the 25th to the 75th percentiles with the median depicted inside the box; whiskers represent the 1st and 99th percentiles.

Comparison between wheat and barley observed and forecasted yields with CORDEX-Evaluation RCM distributions are displayed in Fig. 4 as ECDFs and box plots. After comparing each RCM-derived yield with the observed yield with a two sample Kolmogorov–Smirnov test, the null hypothesis that both samples come from the same distribution is verified, at 5% significance level, for all RCM-derived yields and for the multi-model ensemble. Nevertheless, box plots show the inherent variability among models, notably for wheat in cluster 1.

### Application of the regression models to predict future wheat and barley yields

**Figure 5:**
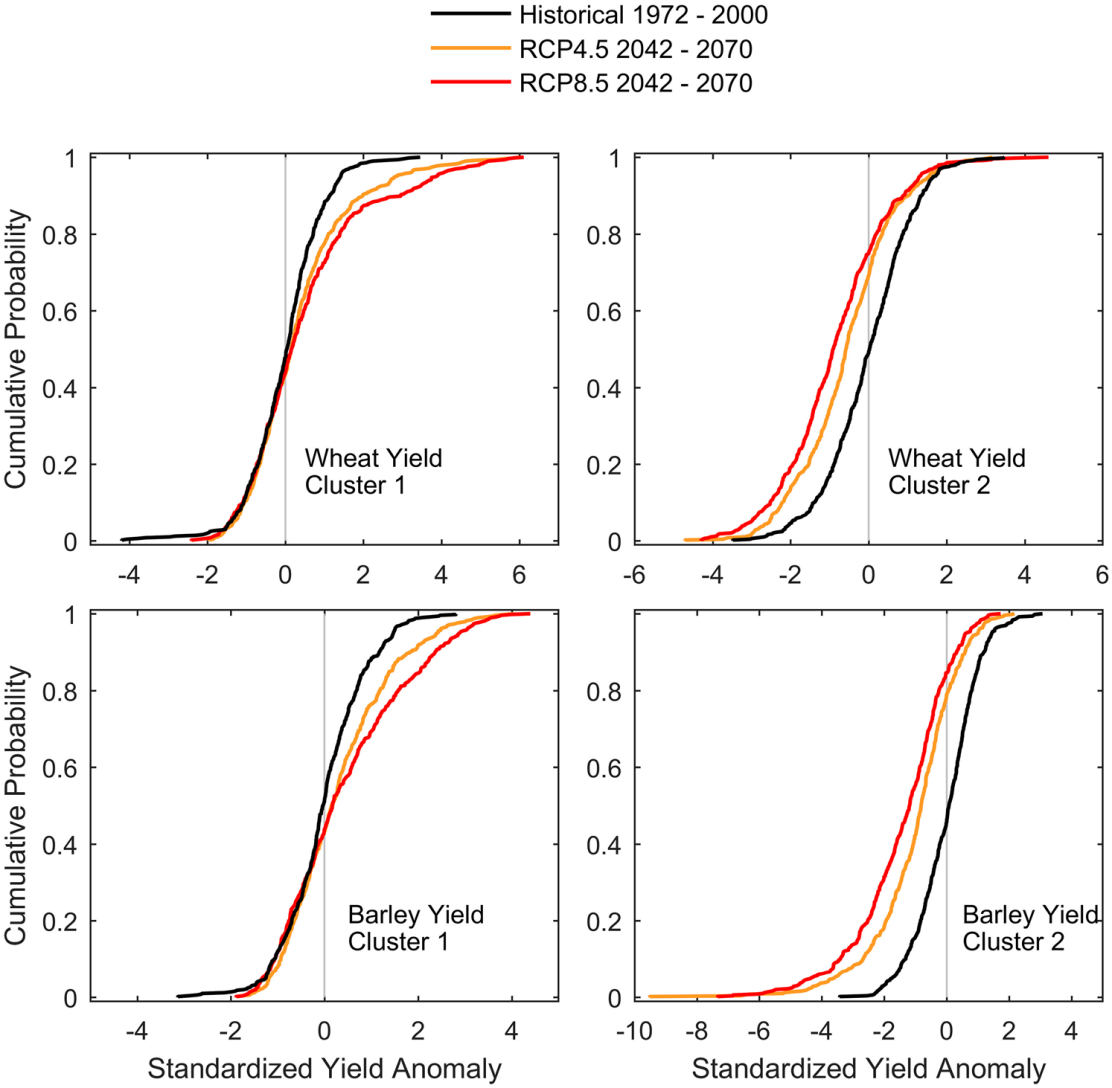
Pooled wheat (top) and barley (bottom) yield anomalies using RCP4.5 (orange) and RCP8.5 (red) for the 2042–2070 period in relation to the historical (1972–2000; black line). Yield was estimated using regression coefficients for individual GCM/RCM models.

Coefficients derived from the Evaluation runs are applied to the GCM forced RCMs for the historical (1972–2000) and mid-of-century (2042–2070) climates (see Supplementary Figure S1 for end-of-century 2071–2100). Figure 5 shows the yield production ECDFs from the multi-model ensemble. Results are similar between cereals, but show different behaviour depending on the cluster. Moreover, Kolmogorov–Smirnov test rejects the null hypothesis that the future (in both RCPs) and historical samples are from the same continuous distribution at 5% significance level.

Over cluster 1, both wheat and barley mid-of-century distributions are similar to the historical yields for negative anomalies, albeit fewer extreme losses are projected. In contrast, positive anomalies in the future differ significantly from the ones estimated for the historical climate, with a propensity to larger positive extremes. Indeed, there is a shift to the right of the ECDFs representing the mid-of-century, which becomes larger with greater yield. With increasing warming, the yield distribution for mid-of-century RCP8.5 shows the largest shift to the right. As an example, wheat yield anomalies larger than 2 have a probability of occurrence of near 0% in the historical period; whereas in the RCP8.5 future climate, this probability increases to about 13%. On the positive extreme of the wheat yield ECDFs spectrum, historical period never displays anomalies larger than circa 3.5, whereas in the mid-of-century these anomalies may surpass 6.

Cluster 2 shows rather different results than those of cluster 1. Here, for both cereals the analysis reveals a shift to the left of the ECDFs representative of the mid-of-century period; i.e., higher probabilities of having yield losses, independently of the anomaly sign. Differences between historical and mid-of-century are more severe than in cluster 1. Indeed, the probability of having barley yield anomaly lower than -2 in the historical climate amounts to about 4% and anomalies smaller than -4 are never identified; in the RCP8.5 (RCP4.5) future climate the probability of having barley yield anomalies lower than -2 reaches more than 30% (18%), and anomaly losses may exceed -7. Notwithstanding, such remarkable shift to the left is not found for positive anomalies, particularly on the extreme end of the ECDFs, where wheat gains remain somewhat similar between historical and mid-of-century climates, and barley anomalies show a decrease from 3 (historical) to 1.8 and 2.2 (RCP8.5 and RCP4.5, respectively).

An additional feature shown by the distributions displayed in Fig. 5 is the propensity for wheat yields to undergo larger future increases than barley yields, over cluster 1; and for barley yields to endure larger future losses than wheat, over cluster 2.

## Discussion

Correlations between climate variables and wheat and barley yields show a very good agreement among RCMs, particularly in terms of precipitation. Here, the growing season cycles show very distinguishable patterns which are in agreement with results presented in another study^53^, stating that a good production year is influenced by precipitation (more pronounced in cluster 2) and warm temperatures (above average in the more temperate north cluster 1, and below average in the drier cluster 2) in late autumn and early winter, which is paramount to prepare the soil to catch-crop. Moderate amounts of precipitation in early spring, more precipitation and below average temperatures in April and May, and a warm and dry June allow for a slow and complete maturation originating filled and well-formed grains^53,54^.

**Figure 6:**
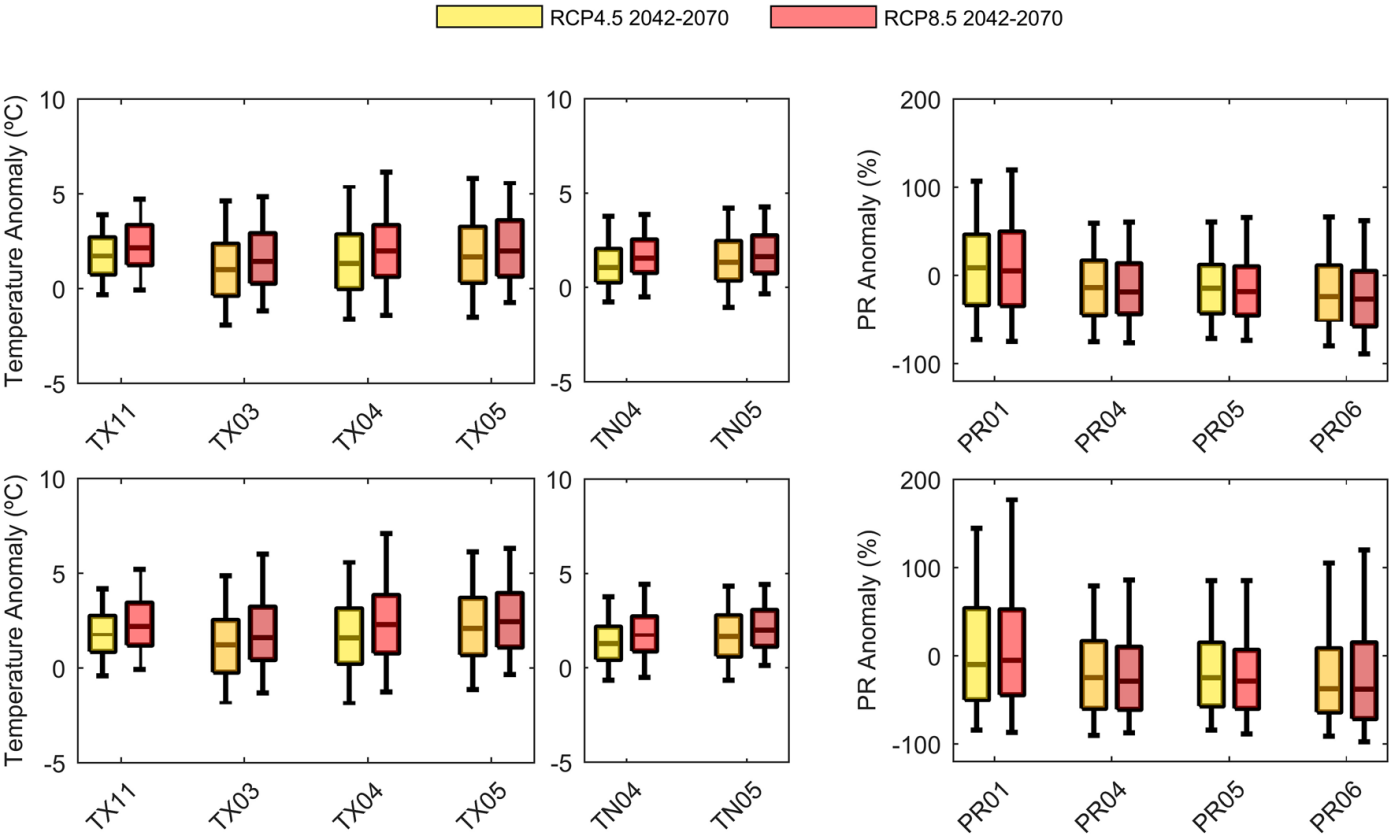
Distribution of TX (°C), TN (°C) and PR (%) anomalies (future–historical) for the most relevant predictors (TX11, TX03, TX04 and TX05; TN04 and TN05; PR01, PR04, PR05 and PR06) for cluster 1 (top) and cluster 2 (bottom). Each boxplot represents the combined distribution of the CORDEX available models for the: (yellow) RCP4.5 2042–2070; (orange) RCP8.5 2042–2070.

The signal of climate change in agreement with the two scenarios for the mid-of-century key selected predictors is illustrated in Fig. 6 (the expanded results with end-of-century are shown in Supplementary Figure S2). These consist of TX in November, March, April, and May; TN in April and May; as well as PR in January, April, May, and June.

In cluster 1, wheat and barley yields are mainly driven by spring precipitation (April and May) with positive coefficient sign. These predictors are expected to have slightly negative anomalies in the future. This would ultimately result in a decrease of cereal yields. However, results show the opposite, with larger yield increases in the mid-of-century. Indeed, other predictors may contribute with an opposite effect, such as TX in November, which is a used predictor in CCLM5-8-17 and RACMO22E for wheat (accounting for circa 40% of the RCMs used), and HIRHAM5 and RACMO22E for barley (almost 35% of the RCMs). Furthermore, changes in TX11 are substantial with respect to those expected in precipitation predictors, which also helps explain such increases in mid-of-century cereal yields.

In cluster 2, spring maximum temperature is the main yield driver (negative coefficients, positive anomalies), which leads to a decrease in both wheat and barley yields in the future.

Indeed, climate change may negatively affect wheat and barley crops situated in regions where temperatures and precipitation are already ideal to its development, where a small change may largely disturb growing mechanisms^55^. On the other hand, the expected warming of colder regions, here exemplified by the northern cluster 1, may have a beneficial outcome in wheat and barley growth, particularly in early winter where higher temperatures may bolster cereal development^56,57^. Furthermore, a comparison between several process-based crop models was previously analysed for barley in the region of Lleida, in the northeaster Spain^58^. In that study, the authors tested the response of crop growth and yield to several perturbed variables, such as temperature, precipitation, solar radiation, and CO_2_. Results showed increased barley yields with an increase in temperature, precipitation, and CO_2_ since a milder winter can be beneficial for the winter barley cultivated there. Such results agree with those presented here for the northern cluster 1. As expected, changes associated with the RCP4.5 scenario are characterized by the same signal and features as RCP8.5, but with smaller magnitudes.

These results focus on heat stress and water deficits at monthly scale, and do not directly account for larger scale extreme climatic events, such as droughts. In fact, droughts may largely contribute to yield variability, particularly in Spain^56^. Indeed, high temperatures are a key driver to increase the vapor pressure deficit (VPD) on the atmosphere and thus the evaporative demand, which in turn contributes to water stress. Hence, drought episodes may exacerbate this process by increasing the water stress^59,60^.

Results from previous studies^42,49^, focusing on the effects of drought, diurnal temperature range, and surface solar radiation to predict the expected range of projected wheat yield trends until the end of the twenty-first century in Spain, agree with those presented here, indicating downward trends in wheat production. Another study^47^ focused on the impact of maximum temperature in maize and spring wheat crops, concluded that southern and northern Spain show dichotomous behaviour, with the southern region being more affected by a decrease in maize yield due to climate change. The projected larger decreases in barley yield when compared with wheat yield concur to the premise that barley is the most vulnerable crop to climate extremes over the provincial clusters analysed in this study which is in accordance with previous studies on that area^61,62^. However, this is still a matter of debate among previous works over the Mediterranean areas^63–66^, and the overperformance of wheat or barley under lowest-yielding conditions is not clear. Nevertheless, few studies have analysed how extreme impacts on both wheat and barley yields are changing under global warming at the sub-national level under rainfed conditions. Other approaches such as the Combined Stress Index (CSI)^67^, which combines Heat Magnitude Day (HMD; dependent on maximum daily temperature) and the Standardized Precipitation-Evapotranspiration Index (SPEI) were used at the country level to estimate climate resilience and anomalies effect on wheat^68^ and maize^69^ in future scenarios of climate change. The CSI has the advantage of depending on non-parametric indicators, i.e., impartial to biases inherent to climate data.

It is important to note that results shown here assume that current wheat and barley genotypes, vernalization, irrigation (or lack thereof), sowing dates, and other management techniques^70^ remain unchanged in the future. Naturally, changes in these factors may be considered adaptation strategies to climate change, which are in continuous development as the impacts and consequences of climate change are more notorious^71–77^. Results presented here assume a scenario of no-adaptation, and thus represent the worst-case hypothesis, which may be softened by adaptation policies. For example, rainfed winter wheat is a difficult crop to implement adaptation measures, being nevertheless possible to effectively adapt by applying a combination of techniques, such as early sowing dates^48^. This may be a viable strategy to overcome climate change impacts in wheat production for the southern cluster, allowing the crop to develop during a cooler period of the year, which may partially avoid water stressed periods^78–80^. Other strategies like double-cropping^81,82^, breeding^83^, adapted crop varieties^84^ may also be applied to mitigate climate change impacts on wheat yields in that region. Conversely, the northward shift of suitable area should make the northern cluster more apt for wheat production, without changing sowing dates^78^. Moreover, it is relevant to stress that projections of wheat and barley yields to the mid-of-century period entail uncertainties due to multiple interrelated biophysical processes^58^. The use of multi-model ensembles should be seen as a tool to reduce these uncertainties. Indeed, the high degree of coincidence between the different GCM-RCM variables implies a robust result. Nevertheless, uncertainties that may arise from model structure and parametrizations of process-based crop models^85^ are not addressed in this work. This is because empirical models do not incorporate a detailed formulation of the physical processes that drive crop and climate relations, aiming only at representing large scale impacts of climate in yields, typically showing similar results to process-based models^52,86^.

This study shows that climate change may have a severe impact on the agricultural landscape as defined today. Indeed, the Iberian Peninsula is notorious for being both a major hot spot for climate change and a major region for rainfed wheat and barley cultivation. However, future changes in cereal production do not show a homogeneous behaviour along the peninsulas’ major farming regions. Indeed, there seems to be a dichotomous demeanour between wheat and barley outputs depending on the latitude they are planted. Severe yield losses are projected for both cereals in the southern region mainly due to the increase in maximum temperatures in spring, particularly when assuming the worst-case scenario of greenhouse gas concentration trajectory. On the other hand, wheat and barley situated further north in the Iberian Peninsula show an increase in yield, which may be linked with the warming of early winter months. This agrees with the observed northward shift of agro-climatic zones and the consequent transition of crop growth suitability^87^. These results show that, as it stands, wheat and barley agriculture may no longer be a viable source of food and income to farmers and to the population in general in southern regions of the Iberian Peninsula, which today largely depends on the social and economic benefits of sowing these cereals. Indeed, this study aims at exposing the influence of climate change (impacts in temperature and precipitation) in the future of these crops, and to alert for the need to implement changes, whether these are local (changing the cultivar species of wheat and barley), country wise (implementing sustainable policies that help mitigate climate change impacts), or even global (mitigate greenhouse gas emissions altogether).

## Methods

Among the Iberian Peninsula provinces with larger percentages of agricultural land-use and dominated by rainfed cropping systems, we selected two clusters for analysis, comprising 10 provinces in Spain. Figure 1 shows the spatial distribution of the provinces which were aggregated in two regionally distinct clusters, each one consisting of 5 neighbouring provinces. The selection criteria of the study area were built on prior works conducted by the authors^35,61^, to which we added the province of Badajoz that has a slightly lower percentage of rainfed area, but similar climate conditions (in terms of precipitation and temperature), allowing therefore to consider the same sample size in each cluster, and hence, making easier an accurate regional comparison between the two clusters.

For the selected provinces, annual yield anomalies (t/ha) of wheat and barley were obtained based on area (ha) and ton of production (t) from the Spanish Agriculture, Food and Environment Ministry (available at https://www.mapa.gob.es). Spatial averages were computed for each cluster and long-term trends were removed by linear detrending, in order to exclude non-climatic factors such as technological development^88^. The time-series were further standardized (Fig. 1, bottom panel).

Precipitation (PR) and daily maximum and minimum temperatures (TX and TN) for the Iberian Peninsula, were retrieved from the EURO-CORDEX 0.11° resolution simulations (Table 1) in the Earth System Grid Federation (ESG) portal. Seven of the 1989 to 2008 ERA-Interim^89^ forced simulations (Evaluation) were used for an initial RCM assessment and the establishment of the regression model predictors. From the 130-year climate projections^26^, 19 GCM-RCM combination results were selected, for the historical period (1971–2000) and two future periods (2041–2070 and 2071–2100), in agreement with the two representative concentration pathways, RCP4.5 and RCP8.5^50^. All simulations were performed on a common European domain and grid.

**Table 1:** CORDEX regional climate models (RCMs) used in this study, along with the forcing and the institute responsible.

Forcing	RCM	Institute	References
ERA-interim	CCLM4-8-17	CLM	Rockel et al.^90^
CNRM-CM5			
EC-Earth			
HadGem2-ES*			
MPI-ESM-LR			
ERA-interim	ALADIN63	CNRM	Nabat et al.^91^
CNRM-CM5			
ERA-interim	HIRHAM5	DMI	Christensen et al.^92^
EC-Earth			
HadGem2-ES*			
NorESM1-M			
ERA-interim	WRF381P	IPSL	Skamarock et al.^93^
IPSL-CM5A-MR			
ERA-interim	RACMO22E	KNMI	Van Meijgaard et al.^94^
CNRM-CM5			
EC-Earth			
HadGem2-ES*			
ERA-interim	REMO2009	MPI	Jacob et al.^95^
MPI-ESM-LR			
ERA-interim	RCA4	SMHI	Samuelsson et al.^96^
CNRM-CM5			
EC-Earth			
IPSL-CM5A-MR			
HadGem2-ES*			
MPI-ESM-LR			
*End-of-century 2072–2098			

Forcings are of two types: (i) ERA-Interim, which represents CORDEX-Evaluation RCMs spanning the period between 1989 and 2008; and (ii) global climate models (GCMs) representing both CORDEX-Historical and CORDEX-Future RCMs. The former describes the present climate (1971–2000), while the latter takes advantage of RCP4.5 and RCP8.5 to describe different future climates.

*End-of-century 2072 - 2098.

In order to study the relationship between the two clusters’ annual production of wheat and barley, and the growing season cycles of TX, TN, and PR, the Pearson's linear correlation coefficient is estimated for each of the seven CORDEX-Evaluation RCMs.

With the aim of predicting wheat and barley production, a stepwise regression is then applied to select sets of statistically significant predictors for linear regression models (the pool of potential predictors consists of monthly TX, TN, and PR from October to June). A total of 28 regression models are developed, representing the seven CORDEX-Evaluation RCMs, two geographic clusters, and two cereals. Overfitting is precluded by using a leave-one-out cross validation scheme^33^. The fitting procedure is repeated by sequentially leaving one observation and the respective predictor(s) out of the training data, and then applying the resulting regression model to the left-out validation data. This approach is repeated by the number of observations that comprise the time-series (19 in this case, representing a given month spanning the 19 years that compose the evaluated period of ERA-Interim forced CORDEX RCMs). The predicted and observed wheat and barley yields with and without cross-validation are subsequently compared using the following metrics: (i) Pearson's linear correlation coefficient R, (ii) the ${\mathrm{R}}^{2}$ adjusted to the number of predictors in the regression model ${\mathrm{R}}_{\mathrm{adj}}^{2}$, (iii) the bias, and (iv) the Mean Absolute Error (MAE). Distributions of predicted and observed wheat and barley yields are displayed as Empirical Cumulative Distribution Functions (ECDFs) and box plots. A two sample Kolmogorov–Smirnov test is used to test the null hypothesis that predicted and observed yields come from the same distribution, at 5% significance level.

For each RCM, the resulting regression model is then applied to estimate wheat and barley yields using the predictors from the respective CORDEX-Historical and CORDEX-Future RCM simulations. Projections of wheat and barley yields for future climates are computed for RCP 4.5 and RCP 8.5 scenarios. Since CORDEX GCM-RCM combinations are numerous, a multi-model ensemble is established by pooling the resulting cereal production yields, i.e., reshaping the 29-year by 18 CORDEX GCM-RCM models’ yield time-series in a one-dimensional sample consisting of 522 forecasted values. ECDFs representative of these pooled yields are further displayed with the objective of identifying potential changes in future wheat and barley production relative to the historical period. To assess if yields derived from CORDEX-Historical and CORDEX-Future are statistically dissimilar, (5% significance level) the nonparametric two-sample Kolmogorov–Smirnov test is used.

To understand the major drivers of yield changing in future climates, anomalies of TX, TN and PR are computed. These are estimated for CORDEX RCMs forced with GCMs for the future relative to the historical climate mean (non-standardized). TX and TN are presented as absolute anomalies (ºC), while PR is displayed as percentage anomalies (%). Box plots of anomalies for the most relevant predictors are displayed.

## Supplementary Information

Supplementary Information.
