# Supplementary material for: The impact of climate change in wheat and barley yields in the Iberian Peninsula
Source: Sci Rep. 2021 Jul 29;11:15484. doi: 10.1038/s41598-021-95014-6 (PMC8322258; doi:10.1038/s41598-021-95014-6)
Supplement: Supplementary file 1 — Supplementary Information. [file 41598_2021_95014_MOESM1_ESM.pdf]

Supplementary Information

When expanding the analysis to the end-of-century period, results of cluster 1 show that mid-of-century and end-of-century RCP4.5 and mid-of-century RCP8.5 climate-derived yields are similar since CO2 emissions until mid-century in RCP4.5 and RCP8.5 scenarios are similar and stabilize until the end of the century in RCP4.5 inducing similar warming between scenarios. With increasing warming, the yield distribution from end-of-century RCP8.5 suggests a larger shift to the right.

For cluster 2, mid- and end-of-century RCP4.5 and mid-of-century RCP8.5 show somehow similar results between them, in order of smallest shift to largest shift to the left: the more conservative pathways of mid- and end-of-century, RCP8.5 mid-of-century, and the most extreme shift by the later period RCP8.5. Differences between historical and future are more severe than in cluster 1.

However, production yields forecasted for the end of the 21st century must be analysed taking into account that statistical models applied to climate projections so far in time may be outside the range of calibration of the MLR predictors defined for the evaluation period, which has unavoidable limitations.

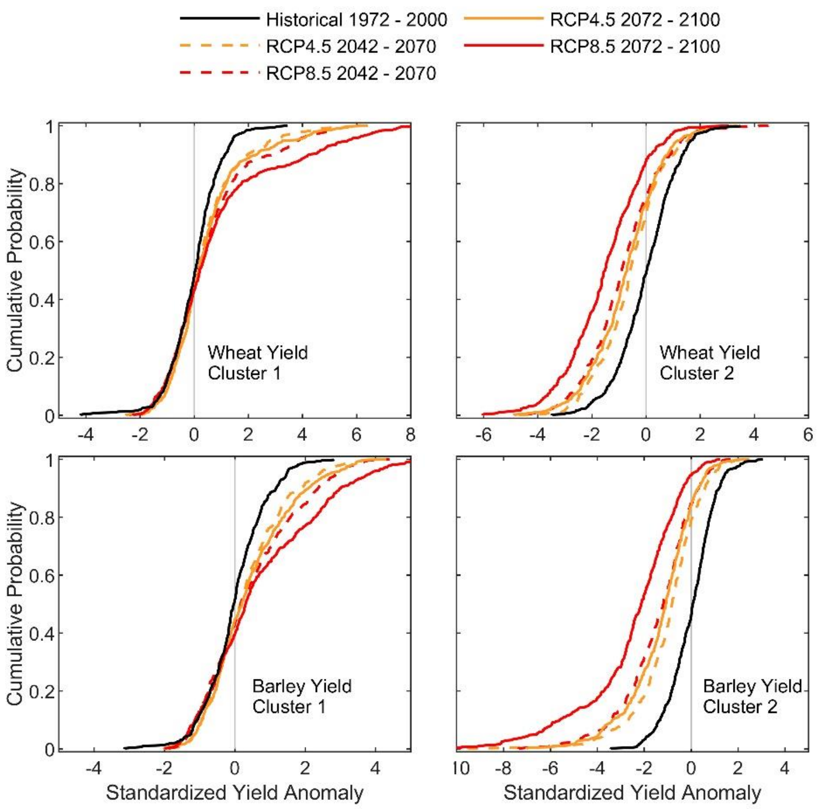

Figure S1 – Wheat (top) and barley (bottom) anomalies using RCP4.5 (dashed) and RCP8.5 (solid lines) for mid-century (2041 – 2060; orange) and end-of-century (2081 – 2100; red) in relation to the control (1986 – 2005; black line). Yield was estimated using regression coefficients for individual GCM/RCM models and then the mean yield presented in the figures was estimated as the arithmetic mean.

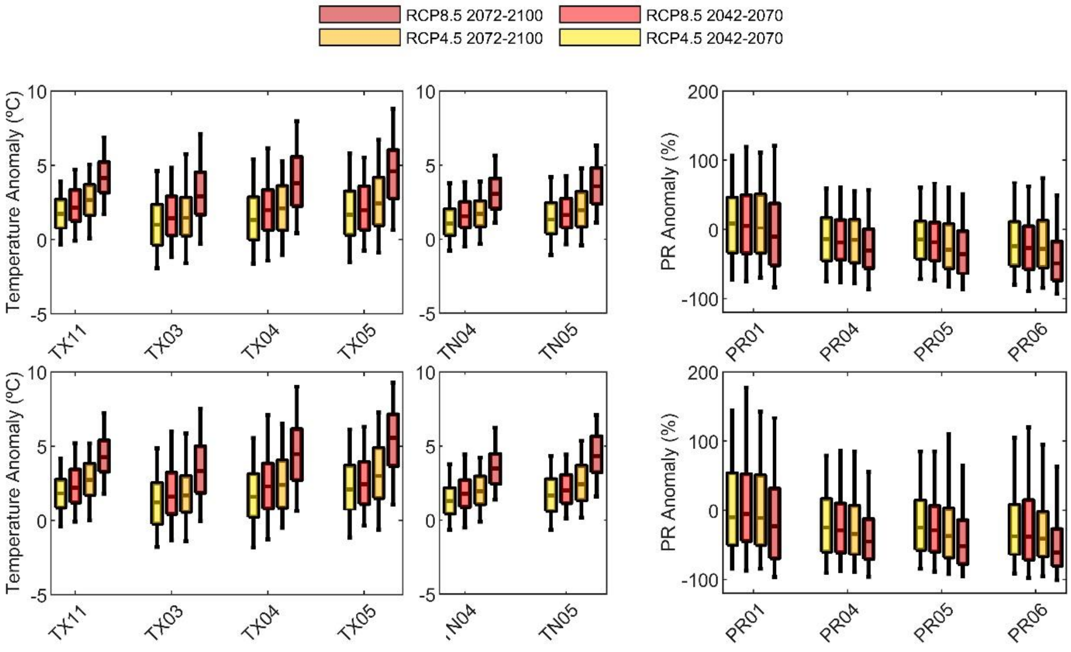

Figure S2 – Distribution of TX (°C), TN (°C) and PR (%) anomalies (future -historical) for the most relevant predictors (TX11, TX03, TX04 and TX05; TN04 and TN05; PR01, PR04, PR05 and PR06) for cluster 1 (top) and cluster 2 (bottom). Each boxplot represents the combined distribution of the CORDEX available models for the: (black) control 1972 – 2000; (yellow) RCP4.5 2042 – 2070; (orange) RCP8.5 2042 – 2070; (dark yellow) RCP4.5 2072 – 2100; and (red) RCP8.5 2072 – 2100.
